# 2057 L1 expression analysis in adipose-derived stem cells

**DOI:** 10.1017/cts.2018.89

**Published:** 2018-11-21

**Authors:** Tiffany Kaul, Rachel Sabol, Maria E. Morales, Bruce Bunnell, Prescott Deininger

**Affiliations:** 1 Center for Stem Cell Research and Regenerative Medicine, Tulane University School of Medicine, New Orleans, LA, USA; 2 Department of Epidemiology, Tulane University Cancer Center, Tulane University, New Orleans, LA, USA

## Abstract

OBJECTIVES/SPECIFIC AIMS: Long interspersed element-1s (L1s) are autonomous, mobile elements that are able to copy and insert themselves throughout the genome with their own reverse transcriptase and endonuclease. These elements make up 17% of the human genome with over 500,000 copies, though the vast majority of L1s are defective with only a few dozen potentially responsible for L1 activity. Full-length L1s have the potential to contribute to mutagenesis through random insertion and increased genetic instability. Here we set out to study L1 expression at the specific loci level in bone marrow-derived stem cells (bmSCs) and adipose-derived stem cells (ASCs) and compare the levels of expression from ASCs from donor patients who are young and lean, obese, and old. Our hypothesis is that L1-related damage may contribute to mutation and inflammation that alters the function of these stem cells throughout the life of an individual. METHODS/STUDY POPULATION: ASCs and bmSCs were isolated from patient donors. The following samples were collected: ASCs from 3 young (under the age of 59) and lean (BMI<30) patients, ASCs from 3 older patients (over the age of 59), ASCs from 3 patients with BMI>30, and bmSCs from 4 young and lean patients. Cytoplasmic RNA from the cell populations was isolated and sequenced by RNA-Seq from the cell populations. Using our recently developed bioinformatics pipeline, we set out to quantify L1 expression and identify the few culprit L1s at specific loci that are actively transcribing to RNA in the ASC and bmSC samples. RESULTS/ANTICIPATED RESULTS: Here we provide proof of concept with the application of this novel method in characterizing full-length expressed L1s at the specific loci level in ASCs and bmSCs. We identified L1 loci that are commonly expressed in these cell types and observed an increase in L1 expression in the obese and old ASC cells compared with the young, lean ASCs and bmSCs. DISCUSSION/SIGNIFICANCE OF IMPACT: ASCs hold the promise of broad application in the biomedical field including regenerative treatment. There are reports that ASCs cultivated from older and obese donors are less effective in regenerative treatments. By demonstrating an increased expression of the mutagenic L1 element in ASCs from obese and old donors, this study provides further evidence suggesting the preferable use of ASCs from young and lean donors for regenerative therapies. These studies will also help us to understand the potential contribution of L1 expression to loss of stem cell function during the aging process.

